# Draft Genome Sequences of Six Skin Isolates of Streptococcus pyogenes

**DOI:** 10.1128/genomeA.00592-18

**Published:** 2018-06-28

**Authors:** Zhong Liang, Melissa Stephens, Victoria A. Ploplis, Shaun W. Lee, Francis J. Castellino

**Affiliations:** a W.M. Keck Center for Transgene Research, University of Notre Dame, Notre Dame, Indiana, USA; b Genomics and Bioinformatics Core Facility, University of Notre Dame, Notre Dame, Indiana, USA; c Department of Chemistry and Biochemistry, University of Notre Dame, Notre Dame, Indiana, USA; d Department of Biological Sciences, University of Notre Dame, Notre Dame, Indiana, USA

## Abstract

Whole-genome shotgun sequences and bottom-up assembly of contigs of six skin isolates of Streptococcus pyogenes, *viz*., NS88.3 (*emm*98.1), NS223 (*emm*91), NS455 (*emm*52), SS1448 (*emm*86.2), SS1572 (*emm*223), and SS1574 (*emm*224), are presented here. All contigs were annotated, and the gene arrangements and the inferred proteins were consistent with a pattern D classification.

## GENOME ANNOUNCEMENT

Group A Streptococcus pyogenes (GAS) is a beta-hemolytic human-pathogenic bacterium. It causes a spectrum of diseases ranging from mild infections to life-threatening tissue destruction and organ failure. This microbe is able to infect multiple human niches, from epithelial surfaces to deeper tissues. Superficial GAS infections at times become highly invasive and result in lethal outcomes if medical intervention does not occur (1).

Herein, we report the newly sequenced bottom-up-assembled contigs of six strains of GAS isolated from skin for future investigations of the pathogenesis associated with skin infection. Isolates NS88.3, NS223, and NS455 were obtained from M. J. Walker (Queensland, Australia), and isolates SS1448, SS1572, and SS1574 were obtained from the Centers for Disease Control and Prevention.

Sequencing libraries were prepared with the NEBNext Ultra II DNA library prep kit (New England BioLabs) and subsequently sequenced across two lanes of an Illumina MiSeq platform using the MiSeq reagent Nano kit version 2 (500 cycles) with 251-bp paired-end reads (Illumina). An average for each genome of 530,000 paired reads passing filtering were produced, yielding ∼135 million bp of data for each of the six GAS strains. Quality control on the library pool was obtained using the Kapa Biosystems quantitative PCR (qPCR) and Agilent Bioanalyzer DNA 7500 assays. Base calling was done by Illumina Real-Time Analysis version 2. The sequencing generated an average of 50-fold coverage for each library, and the sequences were assembled *de novo* using Velvet version 1.2.10 (2). Gene predictions and annotations were obtained using Glimmer 3.0 (3) and RAST (4).

The assembled genomes have an average length of ∼1.76 million bp in ∼34 contigs (range, 26 to 45 contigs), with an average *N*_50_ value of 201,592 ± 41,543 bp. Gene annotations identified an average of 1,730 (range, 1,674 to 1,788) open reading frames. Since gaps between contigs have not as yet been filled, the genes at the termini of the contigs may be fragmented, and small fragments may be absent. Nonetheless, we find that genomic features related to pattern type and virulence exist in all six genomes, including the unique GAS pattern D multiple-gene activator (Mga) arrangement and the type 3 arrangement of the fibronectin-collagen-T-antigen (FCT) locus (5). All six strains also encode the major virulence determinant, *viz.*, the direct plasminogen-binding type M protein (PAM), which is found only in pattern D GAS strains. In the case of strain NS223, PAM is partially present in two contigs, with a missing fragment in the center of the gene. Also of importance, all six isolates contain the streptokinase subtype 2b gene that activates plasminogen bound to PAM-type M proteins (6). Thus, all genomic data obtained to date are consistent with the pattern D skin tropicity of these strains (7–9), similar to the well-characterized genomes of AP53 (9) and Alab49 (10), GAS skin isolates. Identifying the specific virulomes and resistance genes of GAS serotypes by whole-genome sequencing is of great importance in vaccine development and public health approaches to outbreak control.

### Accession number(s).

This whole-genome shotgun project has been deposited at DDBJ/ENA/GenBank under the accession numbers QFXO00000000 (NS88.3), QFXP00000000 (SS1574), QFXQ00000000 (SS1572), QFXR00000000 (SS1448), QFXS00000000 (NS455), and QFXT00000000 (NS223).
